# Preoperative lymphocyte-to-monocyte ratio and CT-measured appendix diameter in occult appendiceal neoplasms: a matched case–control analysis

**DOI:** 10.1186/s12893-025-03328-3

**Published:** 2025-11-21

**Authors:** Yusuf Yunus Korkmaz, Mehmet Şaban Korkmaz, Ayşe Nur Balcı Yapalak, Oğuzhan Aydın, Feyyaz Güngör, İlyas Kudaş, Erdem Kınacı

**Affiliations:** 1 Department of General Surgery, Başakşehir Çam and Sakura City Hospital, Istanbul, Turkey; 2 Deparment of Public Health, Faculty of Medicine, Istanbul Medipol Universty, Istanbul, Turkey

**Keywords:** Appendiceal neoplasm, Lymphocyte‑to‑monocyte ratio, Appendix diameter, Computed tomography, Case-control, Matched analysis

## Abstract

**Introduction:**

Occult appendiceal tumors may present as acute appendicitis, hindering preoperative recognition. Existing clinical and imaging criteria remain nonspecific. This study aimed to identify practical preoperative predictors by integrating hematologic indices with CT morphometry in a matched case–control design.

**Methods:**

In this retrospective matched case–control study, 2,599 adult appendectomy cases (2020–2024) were screened. Sixty-two had histologically confirmed neoplasms; after excluding incomplete records, 40 malignant cases were matched 1:4 by age and sex with 160 benign controls. Preoperative lymphocyte-to-monocyte ratio (LMR) and CT-measured appendix diameter were analyzed using conditional logistic regression.

**Results:**

After matching (SMD < 0.01), malignant cases showed higher LMR (3.41 ± 1.89 vs. 2.51 ± 1.31, *p* = 0.001) and larger diameter (12.6 ± 4.1 mm vs. 10.8 ± 2.7 mm, *p* = 0.001). LMR > 3.8 increased malignancy risk 3.77-fold, diameter > 14 mm increased it 4.9-fold, and both combined raised risk nearly 18-fold (AUC = 0.74; Hosmer–Lemeshow *p* = 0.09).

**Conclusions:**

Elevated LMR and enlarged appendix diameter independently predict occult appendiceal neoplasms. Patients exceeding both thresholds may benefit from intraoperative frozen-section or extended resection. Multicenter validation is warranted.

**Supplementary Information:**

The online version contains supplementary material available at 10.1186/s12893-025-03328-3.

## Introduction

Acute appendicitis is the most common emergency abdominal condition, with a lifetime incidence of 7–8%. However, the incidence of appendiceal neoplasms found in appendectomy specimens ranges between 1 and 3.7% in multicenter studies [1–4]. In the last decade, the appendiceal neuroendocrine tumour subtype has shown a significant increasing trend in cancer registries in the United States, emphasising the clinical importance of overlooked cases [2]. Concordant data from centers with high CT integration, including Turkey, show that the risk of microscopic tumors cannot be ignored even in macroscopically benign tissue [3, 4].

Current guidelines recommend age > 40 years, appendix diameter > 10 mm, and luminal obstruction as ‘red flag criteria’, but the specificity of these signs remains low and has been shown to detect only one in three cases [5, 6]. In the same period, non-operative treatment (NOT) protocols have been increasingly adopted in the management of acute appendicitis, and the prevalence of neoplasia in interval appendectomies is reported to be up to 11% [7]. This rate is approximately 4–10 times higher compared to the incidence of 1–3.7.7% in series with primary surgery in the acute phase; therefore, selective secondary surgery should be discussed in patients for whom the NOT algorithm has been selected in order not to miss the silent tumor [8]. Therefore, whichever algorithm, surgical or conservative, is chosen, there remains a need for accurate risk models that integrate cheap and easily accessible biomarkers with imaging findings.

While normal or low white blood cell counts (WBC) and a low neutrophil-to-lymphocyte ratio (NLR) have been associated with malignancy in some studies, the results are inconsistent [9, 10]. The lymphocyte-to-monocyte ratio (LMR), which has been repeatedly validated as a prognostic marker in solid organ cancers, has not been systematically tested in the acute surgical population [11]. CBC parameters with appendix diameter to produce a simple score based on ‘haemogram + morphometry’ points to an important gap in the literature.

Appendiceal neoplasms can present with features indistinguishable from acute appendicitis, often resulting in delayed diagnosis and suboptimal management. Therefore, this study aimed to investigate the association between preoperative hematologic indices derived from complete blood count and CT-measured appendix diameter with histologically confirmed appendiceal neoplasms in a matched case–control design.

This exploratory approach may contribute to defining discriminative preoperative markers that can improve awareness of occult neoplasms during the management of acute appendicitis.

## Methods

This study aimed to evaluate the association between preoperative complete blood count (CBC) indices and CT-measured appendix diameter with histologically confirmed appendiceal neoplasms. The study was designed as a retrospective, matched case–control analysis conducted at Istanbul Başakşehir Çam and Sakura City Hospital between 1 May 2020 and 30 April 2024. Given the rarity of appendiceal neoplasms, age- and sex-frequency matching (1:4) was applied to ensure demographic balance and stabilize parameter estimation within matched strata. Reporting was performed in accordance with STROBE guidelines [12].

A total of 2599 cases were analyzed. Patients operated for indications other than acute appendicitis, patients under 18 years of age, and files with missing age, sex, complete blood count (CBC), preoperative computed tomography (CT) report, or postoperative histopathology data were excluded. The following variables were systematically recorded from the electronic archive: age, sex, laboratory parameters [white-blood-cell count (WBC), neutrophil, lymphocyte, monocyte, platelet, eosinophil], derivative indices [neutrophil-to-lymphocyte ratio (NLR), platelet-to-lymphocyte ratio (PLR), lymphocyte-to-monocyte ratio (LMR), systemic immune-inflammation index (SII)] and C-reactive protein (CRP). Preoperative CT reports, appendix diameter measurement, periappendicular inflammation, abscess and perforation findings, and postoperative histopathological results were also recorded. Of the 62 histologically confirmed neoplasia cases, 22 were excluded due to missing preoperative CBC or CT data. We examined the missingness mechanism and found no significant differences in age or sex between excluded and included cases, indicating missing completely at random (MCAR). Therefore, the exclusion is unlikely to have introduced systematic bias.

Preoperative abdominal CT images were retrospectively analyzed from the hospital PACS archive. Two expert general surgeons blinded to the histopathological results measured the appendix diameter independently by determining the widest point in the axial plane. When the difference between the measurements was > 2 mm, the sections were evaluated and reconciled together; the final value was recorded as the mean of the two measurements. Periappendicular inflammation, abscess, and perforation findings were binary coded as ‘present’/‘absent’. Inter-observer agreement between the two raters was excellent (ICC = 0.94; 95% CI 0.89–0.97).

Histopathological classification of ‘neoplasia’ was based on the criteria in the World Health Organization (WHO) Classification of Tumours of the Digestive System, 5th edition; the definition was expanded to include all subtypes of epithelial tumors, neuroendocrine tumors, and low-grade appendiceal mucinous neoplasms (LAMN) [13].

For each neoplasia case, four benign controls of the same sex and ± 2 years of age were selected to achive demographic balance (1:4 age- and sex-frequency matching). Controls were randomly sampled from the study population to form a single ‘block’ for each case; block identities were used as strata(match_id) variables in statistical analysis.

Statistical analyses were performed in IBM SPSS Statistics v24 and R v4.3.2. The distribution of continuous variables was assessed using the Kolmogorov-Smirnov test; normally distributed data were reported as mean ± SD, and non-normally distributed data as median. Student t or Mann-Whitney U test was used as appropriate for group comparisons, and Pearson chi-square or Fisher exact test was used for categorical variables.

A conditional logistic regression model was established for the association analysis, preserving the age-sex matched strata. Variables exceeding *p* < 0.20 in the univariate stage were selected into the multiple models with 10-fold cross-validated Lasso (α = 1). Adjusted odds ratio (OR) and 95% confidence intervals were reported in the final model; model discrimination power was assessed by area under the ROC curve (AUC) and concordance statistic (c-index), calibration by Hosmer-Lemeshow goodness-of-fit test, and overall error by Brier score. The 2 000-bootstrap method was applied for internal validation.

ROC analysis was performed with the pROC package; optimum cut-off points for LMR, appendix diameter, and NLR were determined using the Youden index. Binary variables derived from these cut-off points were tested in a second binary cut-off model. In all analyses, two-way *p* < 0.05 was accepted as the statistical significance threshold.

Ethical approval was obtained from the Clinical Research Ethics Committee of Başakşehir Çam and Sakura City Hospital (No: 2024-104, 10 July 2024). The study complies with the Declaration of Helsinki; patient identity data were concealed, and all analyses were performed with anonymized data. The requirement for informed consent was waived by the Clinical Research Ethics Committee of Başakşehir Çam and Sakura City Hospital in accordance with national regulations and the retrospective design of the study.

## Results

**Fig. 1:**
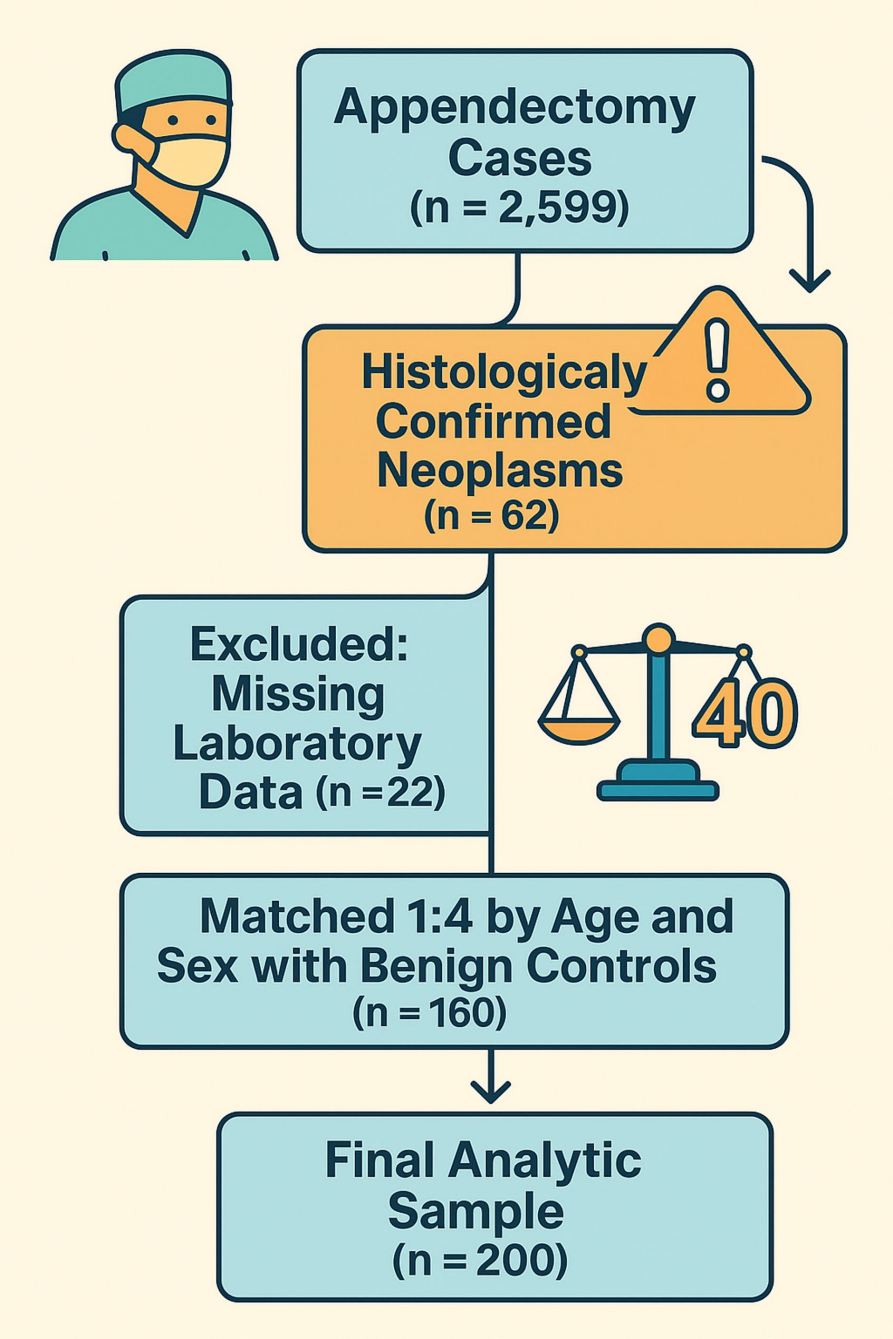
STROBE-style flow diagram of patient selection and matching. Visual summary of the study cohort. Among 2 599 consecutive appendectomy specimens, 62 occult appendiceal neoplasms were identified. Twenty-two cases lacked complete laboratory data and were excluded. The remaining 40 malignant cases were matched to 160 benign controls in a 1 : 4 ratio based on age and sex, yielding a final analytic sample of 200 patients

Neoplasms were detected by postoperative histopathology in 2.3% (*n* = 62) of the 2 599 appendectomy cases examined during the study period. In the neoplasm group, the female rate was 54.8% (*n* = 34), and the male rate was 45.2% (*n* = 28), and the gender distribution was significantly different (χ², *p* = 0.018). The mean age increased from 33.7 ± 12.7 years in benign cases to 43.1 ± 14.2 years in the neoplasm group; the age difference was statistically significant (*p* < 0.001). Due to incomplete laboratory data, 22 neoplasm cases were excluded from the analysis. For the remaining 40 neoplasm cases, 160 benign controls were selected using 1:4 age- and sex-based frequency matching, resulting in a final analytic cohort of 200 patients. Standardized mean difference (SMD) values for age and sex after matching were 0.002 and < 0.001, respectively; these values were below the < 0.10 cut-off, indicating that demographic balance was successfully achieved. The sample flow is summarised in Fig. 1.

### Baseline clinical and laboratory characteristics

It was confirmed that age and sex distributions remained balanced in the matched case-control of 200 subjects (*p* = 1.000; SMD < 0.01). Malignant cases exhibited significantly higher Lymphocyte-to-Monocyte Ratio (LMR) [3.41 ± 1.89 vs. 2.51 ± 1.31; *p* = 0.001; SMD = 0.553] and larger appendix diameter [12.59 ± 4.06 mm vs. 10.81 ± 2.74 mm; *p* = 0.001; SMD = 0.514] compared to benign controls. In contrast, white blood cell count (WBC) and absolute neutrophil count were significantly lower in the malignant group (*p* = 0.029 and *p* = 0.038, respectively). Neutrophil-to-Lymphocyte Ratio (NLR) also showed a lower value in the malignant group (*p* = 0.032), but the effect size was moderate (SMD = 0.411). No significant difference was observed between the two groups in terms of thrombocyte indices and C-reactive protein (CRP) levels. Detailed values are presented in Table 1.

**Table 1:** Baseline characteristics of the age- and sex-matched cohort (*n* = 200)

Variable	Benign (*n* = 160)	Malign (*n* = 40)	*p*-value	SMD
Age, years	42.32 ± 15.71	42.33 ± 15.86	1.000	< 0.001
Sex, female/male, n (%)	88 (55)/72 (45)	22 (55)/18 (45)	1.000	< 0.001
WBC (10³ µL⁻¹)	12.63 ± 3.73	11.15 ± 4.05	0.029	0.379
Neutrophils (10³ µL⁻¹)	9.75 ± 3.58	8.41 ± 3.92	0.038	0.358
Lymphocytes (10³ µL⁻¹)	1.91 ± 0.88	2.08 ± 0.74	0.240	0.219
Platelets (10³ µL⁻¹)	250.07 ± 61.43	258.60 ± 89.74	0.478	0.111
Monocytes (10³ µL⁻¹)	0.93 ± 1.16	0.73 ± 0.34	0.305	0.223
Eosinophils (10³ µL⁻¹)	0.09 ± 0.12	0.09 ± 0.08	0.990	0.002
NLR	6.78 ± 5.01	4.94 ± 3.86	0.032	0.411
PLR	160.46 ± 91.10	142.56 ± 83.02	0.260	0.205
LMR	2.51 ± 1.31	3.41 ± 1.89	0.001	0.553
SII	1 634.70 ± 1 202.56	1 270.84 ± 1 079.50	0.082	0.318
CRP, mg L⁻¹	61.96 ± 86.31	55.33 ± 61.53	0.648	0.088
10.81 ± 2.74	12.59 ± 4.06	0.001	0.514	
Peri-appendicular inflammation	0.64 ± 0.48	0.55 ± 0.50	0.276	0.190
Perforation sign	0.06 ± 0.24	0.02 ± 0.16	0.355	0.183
Abscess sign	0.04 ± 0.21	0.02 ± 0.16	0.591	0.102

Values are mean ± SD for continuous variables and *n* (%) for categorical variables. *p*-values derive from conditional logistic regression, *SMD * standardised mean difference

Abbreviations: *WBC* white blood cell count, *Neutrophils* absolute neutrophil count, *Lymphocytes*, absolute lymphocyte count, *Platelets* absolute platelet count, *Monocytes* absolute monocyte count, *NLR* neutrophil-to-lymphocyte ratio, *PLR* platelet-to-lymphocyte ratio, *LMR* lymphocyte-to-monocyte ratio, *SII* systemic immune-inflammation index, *CRP* C-reactive protein

### Univariable analysis

**Fig. 2:**
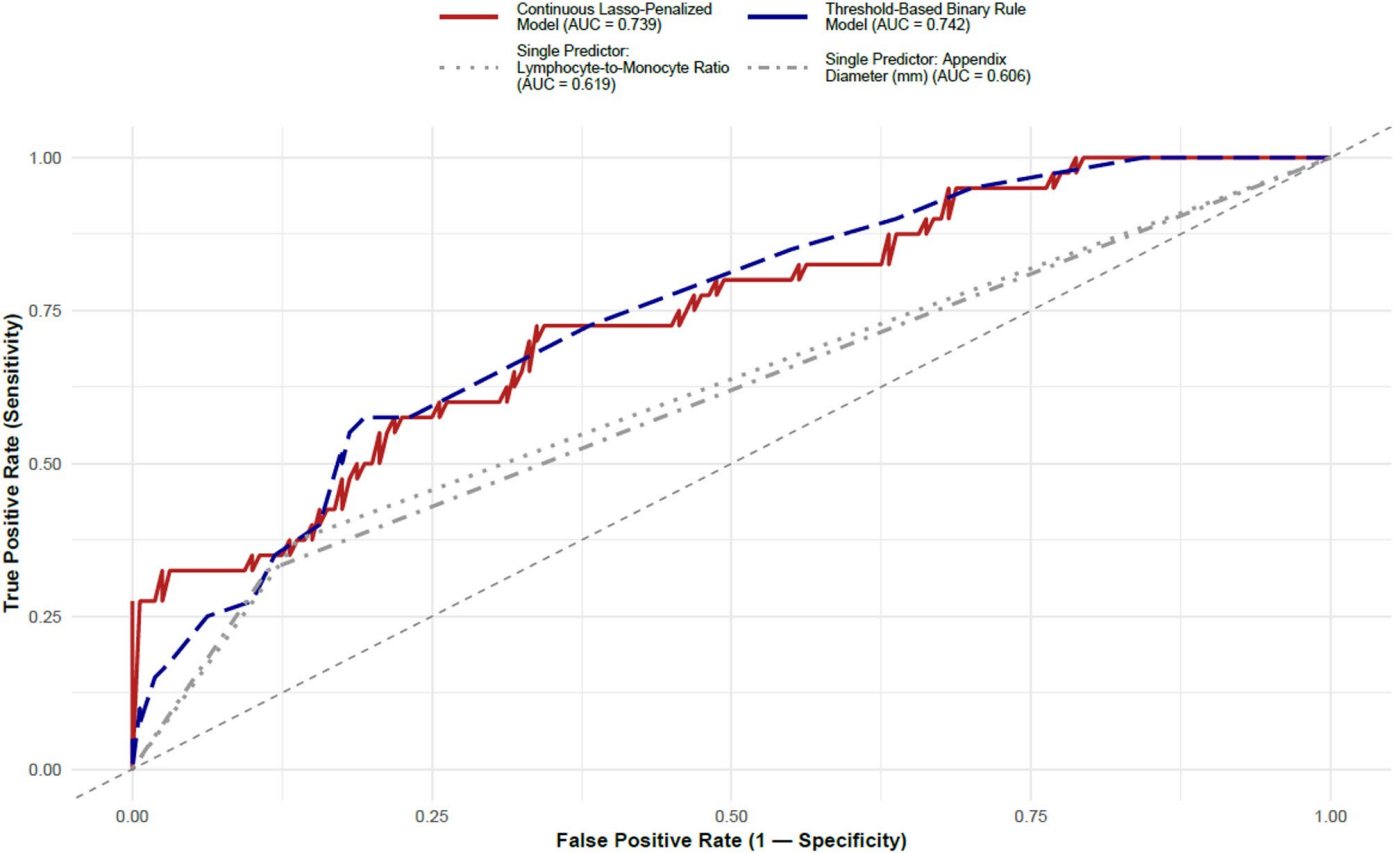
Receiver‑operating characteristic curves for models predicting occult appendiceal neoplasms. Receiver‑operating characteristic (ROC) analysis comparing four pre‑operative prediction approaches. The continuous Lasso‑penalised multivariable model that integrates lymphocyte‑to‑monocyte ratio (LMR) and CT‑measured appendix diameter (red solid line) achieved an AUC of 0.739 (95% CI 0.656–0.821). A simple threshold‑based binary rule that flags patients when LMR > 3.78 and/or appendix diameter > 14 mm (blue dashed line) yielded a similar AUC of 0.742 (95% CI 0.657–0.817). Single‑predictor models displayed lower discrimination: LMR alone (dark‑grey dotted line, AUC 0.619) and appendix diameter alone (light‑grey dash‑dot line, AUC 0.606). The grey 45° diagonal represents no discrimination

Conditional logistic regression preserving age–sex matching identified lymphocyte-to-monocyte ratio (LMR) and appendix diameter as independent variables associated with appendiceal neoplasia. Conversely, WBC count, absolute neutrophil count, and neutrophil-to-lymphocyte ratio (NLR) were inversely associated with neoplasia. ROC analysis indicated that cut-off values of LMR > 3.78 and appendix diameter > 14.1 mm achieved the most favorable sensitivity–specificity balance **(**Table 2; Fig. 2**).**Conditional logistic regression preserving age–sex matching identified lymphocyte-to-monocyte ratio (LMR) and appendix diameter as independent variables associated with appendiceal neoplasia. Conversely, WBC count, absolute neutrophil count, and neutrophil-to-lymphocyte ratio (NLR) were inversely associated with neoplasia. ROC analysis indicated that cut-off values of LMR > 3.78 and appendix diameter > 14.1 mm achieved the most favorable sensitivity–specificity balance **(**Table 2; Fig. 2**).**

**Table 2:** Univariable matched conditional logistic regression and ROC metrics (*n* = 200)

Predictor	Cut-off	OR	95% CI	*p*-value	AUC (95% CI)	Sens	Spec
LMR	> 3.78	1.43	1.15–1.79	0.002	0.643 (0.55–0.73)	38%	86%
> 14.1	1.20	1.07–1.35	0.002	0.629 (0.54–0.72)	33%	89%	
WBC (10³ µL⁻¹)	≤ 9.32	0.90	0.81–0.99	0.034	0.604 (0.51–0.70)	40%	80%
Neutrophils (10³ µL⁻¹)	≤ 8.16	0.90	0.81–1.00	0.044	0.601 (0.50–0.69)	60%	70%
NLR	≤ 3.68	0.90	0.81–1.00	0.039	0.632 (0.54–0.72)	60%	70%

*Both binary predictors included simultaneously,†HL* p*-value calculated with five risk groups due to sparse deciles

*OR*odds ratio,* CI*confidence interval,* AUC*area under the ROC curve,* Sens*sensitivity,* Spec*specificity,* LMR*lymphocyte-to-monocyte ratio,* NLR*neutrophil-to-lymphocyte ratio,*WBC*white blood cell count

### Multivariable model

LMR and appendix diameter remained independently associated with appendiceal neoplasia in the conditional logistic regression model (Table 3; Fig. 2). The continuous model showed good discriminative performance (AUC = 0.739; 95% CI 0.656–0.821) and satisfactory calibration (Hosmer–Lemeshow *p* = 0.316). The binary model, constructed using ROC-derived thresholds, demonstrated comparable discrimination (AUC = 0.742; 95% CI 0.657–0.817) and consistent model fit (Hosmer–Lemeshow *p* = 0.091). For the threshold-based binary model (LMR > 3.78; diameter > 14 mm), the bootstrap-corrected calibration plot similarly showed low deviation (mean absolute calibration error = 0.018; 0.9 quantile = 0.032), with a Brier score of 0.227 (95% CI 0.204–0.245) and satisfactory discrimination (AUC = 0.742; 95% CI 0.657–0.817). These results confirm that both models maintained acceptable calibration and discrimination performance.

**Table 3:** Multivariable matched conditional logistic regression and model performance (*n* = 200)

Model	Predictor/metric	OR	95% CI	*p*-value	AUC (95% CI)	HL *p*	Brier
Continuous predictors	LMR (per unit)	1.55	1.22–1.96	< 0.001	0.739 (0.656–0.821)	0.316	0.184
	1.27	1.10–1.46	0.001				
Binary cut-off model*	LMR > 3.78	3.77	1.64–8.66	0.002	0.742 (0.657–0.817)	0.091†	0.186
	4.85	1.82–13.00	0.002				

*Both binary predictors included simultaneously,†HL *p*-value calculated with five risk groups due to sparse deciles

*OR*odds ratio,*CI*confidence interval,*AUC*area under the ROC curve,*HL*Hosmer–Lemeshow goodness-of-fit test,*LMR*lymphocyte-to-monocyte ratio

## Discussion

This matched case-control analysis demonstrates that an increase in appendix diameter and an elevated lymphocyte-monocyte ratio (LMR) in patients undergoing appendectomy for appendicitis are independently associated with appendix neoplasia. The malignancy rate observed in our study was 2.3% (62/2,599), consistent with the range of 0.7–3.7% reported in recent systematic reviews and multicenter cohorts [1, 2, 4]. Given the limited number of malignant appendiceal cases in the present cohort (*n* = 40), subgroup analyses by histological subtype were not performed, as such stratification would have compromised statistical reliability and interpretability. Although the malignant group included various histopathological entities—including neuroendocrine tumors, low-grade appendiceal mucinous neoplasms (LAMN), and adenocarcinomas—these lesions share a comparable chronic pathological background and often present with similar clinical manifestations when mimicking acute appendicitis. For this reason, all malignant cases were analyzed as a single group to preserve statistical robustness while allowing meaningful comparison with benign controls. This analytical choice aligns with previously published single-center series addressing rare appendiceal malignancies.

A recent meta-analysis combining seventeen studies (*n* = 10 487) reported an overall incidence of appendiceal neoplasia of 1.9% (95% CI 1.3–2.6%), highlighting wide inter-centre heterogeneity [4]. Two series, one originating in Latin America (*n* = 2 993) and the other in Switzerland (*n* = 457), found an incidence of 2.1% and 3.7%, respectively; these values support the incidence in our study [1, 2]. These findings emphasize the importance of identifying accessible and practical preoperative markers associated with appendiceal neoplasia.

Although the antibiotic-led conservative approach to acute appendicitis is becoming increasingly popular, the risk of missing the underlying neoplasia should not be ignored. In the literature, the rate of occult tumour in emergency appendectomy specimens has been reported between 0.7% and 2.5% [5, 14, 15]. This risk is higher in patients who are followed conservatively: Sceats et al. (Non-operative treatment in perforated appendicitis; *n* = 436) found a 2.5% rate of late diagnosed neoplasia [16]. Ramadan et al. reported a 5% (12/259) rate of malignancy in complicated appendicitis in a series of 259 patients [17]. In a pooled systematic review of 8 studies evaluating interval appendectomies following conservative treatment, the prevalence of neoplasia was 11% (CI 95% 7–15%) [7]. This high prevalence once again emphasises the importance of non-invasive predictive markers for the early diagnosis of appendiceal neoplasia.

Age and appendix diameter consistently appeared as the most relevant anatomical parameters. Brunner et al. reported that the combination of ≥ 13 mm diameter on ultrasonography and age ≥ 50 years was associated with appendiceal neoplasia (AUC 0.63) [5]. An American cohort of 440 patients showed that neoplasia was more frequent in those > 40 years old with CT-measured appendix diameter > 10 mm, while Loftus et al. also linked older age and steroid/immunosuppressive use with tumor presence [6, 14]. In our matched case–control design, age and sex effects were minimized due to 1:4 age–sex matching. Despite this, the association between increased appendix diameter and neoplasia remained significant (aOR 1.27/mm; *p* = 0.001), indicating that a wider lumen may be associated with neoplasia independent of demographic variables.

Previous studies evaluating blood-based indices have yielded conflicting results. Stopenski et al. reported an association between low leukocyte count (WBC < 10 × 10³/µL) and appendiceal neoplasia (OR: 1.58). This observation may indicate that a limited inflammatory response reflects the atypical clinical presentation often seen in neoplastic processes. Koç et al. also found lower WBC and neutrophil counts among patientes wtih tumor cases [9]. These findings are consistent with the lower leukocyte and neutrophil counts observed in our study.

The novelty of this study lies in being the first quantitatively demonstrate an independent association between lymphocyte-to-monocyte ratio (LMR) and appendiceal neoplasm. In conditional logistic regression model, each 1-unit increase in LMR was associated with higher odds of neoplasia (adjusted OR 1.55; 95% CI 1.21–1.99; *p* = 0.001). Likewise, patients with LMR values above the ROC-derived thresold of 3.78 showed higher odds of neoplasia (adjusted OR 3.77; 95% CI 1.64–8.66; *p* = 0.002). Although the association between LMR and prognosis has been described in various solid tumours, its quantitative relationship with appendiceal lesions is demostrated here fort he first time [11]. Mechanistically, the relative increase in monocytes during chronic intraluminal inflammation and limited acute phase response may reflect an altered lymphocyte/monocyte balance contributing to neoplastiv processes.

In contrast, although NLR is frequently used as an indicator of severity in complicated appendicitis, it was not retained in our multivariate model. Ishizuka et al. reported that NLR > 8 was associated with gangrenous appendicitis, but this relationship does not extend to neoplasia [15]. Interestingly, Pedük et al. observed higher NLR values(OR 1.21) to be independently associated with appendiceal neoplasia in a series of 598 patients [16]. These contrasting findings may be explained by population differences or the addition of clinical variables such as Hb and Alvarado scores to the model in the Peduk study. Therefore, the relationship between NLR and appendiceal neoplasia remains controversial, and our results suggest that LMR may represent a more consistent hematologic marker in this context.

The AUC of our two-variable conditional logistic regression model (appendix diameter and LMR) was 0.739, which showed higher discriminative ability than Brunner’s multivariable analysis (AUC 0.63) [5]; and also exceeded single-parameter analyses reported in smaller cohorts (AUC 0.55–0.66) [9, 17]. The model including appendix diameter > 14.1 mm and LMR > 3.78 demonstrated comparable discriminative performance (AUC 0.742) and good calibration (Hosmer–Lemeshow *p* = 0.091). Our diameter threshold (> 14.1 mm) aligns with multicenter analyses reporting approximately 3-fold higher odds of neoplasia in patients aged ≥ 40 years with CT-measured appendix diameter > 10 mm, and with classical CT series indicating that diameter > 15 mm is typical for primary appendiceal tumors [6, 18]. Since no standardized LMR cut-off has been defined in prior literature, external validation of these associations is required.

Neuroendocrine tumors smaller than 2 cm and without mesoappendiceal invasion can be cured with appendectomy alone, while right hemicolectomy is recommended for tumors ≥ 2 cm in diameter or with high-risk features [19]. LAMN cases should be investigated for acellular mucin or pseudomyxoma peritonei; early oncological referral is mandatory for these patients. The identified associations may contribute to earlier multidisciplinary evaluation by highlighting preoperative features linked with neoplasia. These findings are exploratory and may help refine future criteria for surgical decision-making once validated in larger, prospective cohorts. This approach may ultimately assist in avoiding unnecessary extensive surgery while ensuring oncologic completeness in patients who genuinely require extended resection.

As recommended by the STROBE framework for rare outcomes, the use of 1:4 frequency matching case-control helped control for major confounders such as age and sex, thereby enhancing internal validity, narrowing confidence intervals, and supporting the high inter-observer agreement (ICC = 0.94) for diameter measurements.

### Clinical relevance and limitations

The associations identified between LMR and appendix diameter may have practical implications for surgical decision-making in patients with suspected appendiceal neoplasia.When both parameters are elevated (LMR > 3.8 and appendix diameter > 14 mm), these findings may alert surgeons to the potential presence of neoplastic pathology, encouraging careful intraoperative assessment and consideration of frozen-section evaluation if necessary.

In contrast, the widespread adoption of non-operative treatment (NOT) for acute appendicitis should be approached cautiously in such patients, as unrecognized neoplasia may contribute to delayed diagnosis and adverse outcomes.

The study’s retrospective, single-center design and the relatively limited number of malignant cases (*n* = 40) precluded detailed subgroup analyses by histologic subtype. Additionally, molecular and radiomic parameters were not assessed. The relatively high exclusion rate within the malignant group (22 of 60 initially identified cases) primarily reflected technical constraints rather than biological or demographic factors. Most exclusions were due to incomplete or low-resolution CT datasets that precluded reliable measurement of appendiceal diameter, along with a small number of cases lacking complete laboratory records. Since these omissions were unrelated to patient characteristics or disease severity, the missing data were categorized as *missing at random (MAR)*. Although this reduction in sample size may have modestly decreased statistical power, it is unlikely to have introduced systematic selection bias or altered the direction of the observed associations. Nevertheless, this limitation has been acknowledged as a potential source of reduced generalizability, underscoring the need for larger, multicenter datasets to validate the present findings. The relatively wide confidence intervals observed for some associations, including that between LMR > 3.78 and appendiceal neoplasms, likely reflect the limited sample size rather than measurement variability. Future multicenter case–control studies with larger malignant cohorts are warranted to confirm the strength and consistency of these associations.

Future prospective, multicenter studies are warranted to validate the observed associations and to integrate laboratory, imaging, and molecular variables into a more comprehensive diagnostic framework.

## Conclusion

This matched case–control study demonstrates that preoperative lymphocyte-to-monocyte ratio (LMR) and CT-measured appendix diameter are independently associated with occult appendiceal neoplasms.These two easily obtainable parameters showed good discriminative performance in differentiating neoplastic from benign cases.The identified thresholds (LMR > 3.8 and appendix diameter > 14 mm) may serve as reference values to enhance preoperative awareness of occult neoplasia.

## Supplementary Information

Supplementary Material 1.

## Data Availability

The datasets generated and/or analysed during the current study are available from the corresponding author on reasonable request.
