# Supplementary material for: Preoperative lymphocyte-to-monocyte ratio and CT-measured appendix diameter in occult appendiceal neoplasms: a matched case–control analysis
Source: BMC Surg. 2025 Nov 21;25:563. doi: 10.1186/s12893-025-03328-3 (PMC12639642; doi:10.1186/s12893-025-03328-3)
Supplement: Supplementary file 1 — Supplementary Material 1. [file 12893_2025_3328_MOESM1_ESM.docx]

# Supplementary Table S1. Univariable matched conditional logistic regression for appendiceal malignancy (n = 200)

| Predictor | OR | 95 % CI | p-value |
| --- | --- | --- | --- |
| **LMR** | **1.43** | **1.15 – 1.79** | **0.002** |
| **Appendix diameter (mm)** | **1.20** | **1.07 – 1.35** | **0.002** |
| WBC (10³/µL) | 0.90 | 0.81 – 0.99 | 0.034 |
| NLR | 0.90 | 0.81 – 1.00 | 0.039 |
| Neutrophils (10³/µL) | 0.90 | 0.81 – 1.00 | 0.044 |
| Monocytes (10³/µL) | 0.34 | 0.10 – 1.13 | 0.079 |
| SII | 1.00 | 0.999 – 1.00 | 0.093 |
| Lymphocytes (10³/µL) | 1.29 | 0.85 – 1.95 | 0.226 |
| Peri-appendicular inflammation | 0.65 | 0.31 – 1.36 | 0.258 |
| PLR | 1.00 | 0.99 – 1.00 | 0.271 |
| Perforation sign | 0.38 | 0.05 – 3.07 | 0.361 |
